# A Single 48 mg Sucralose Sip Unbalances Monocyte Subpopulations and Stimulates Insulin Secretion in Healthy Young Adults

**DOI:** 10.1155/2019/6105059

**Published:** 2019-04-28

**Authors:** Angélica Y. Gómez-Arauz, Nallely Bueno-Hernández, Leon F. Palomera, Raúl Alcántara-Suárez, Karen L. De León, Lucía A. Méndez-García, Miguel Carrero-Aguirre, Aaron N. Manjarrez-Reyna, Camilo P. Martínez-Reyes, Marcela Esquivel-Velázquez, Alejandra Ruiz-Barranco, Neyla Baltazar-López, Sergio Islas-Andrade, Galileo Escobedo, Guillermo Meléndez

**Affiliations:** ^1^Laboratory for Proteomics and Metabolomics, Research Division, General Hospital of Mexico “Dr. Eduardo Liceaga”, 06720 Mexico City, Mexico; ^2^Clinical Nutrition Division, General Hospital of Mexico “Dr. Eduardo Liceaga”, 06720 Mexico City, Mexico; ^3^Research Coordination at Central Laboratories, General Hospital of Mexico “Dr. Eduardo Liceaga”, 06720 Mexico City, Mexico

## Abstract

Sucralose is a noncaloric artificial sweetener that is widely consumed worldwide and has been associated with alteration in glucose and insulin homeostasis. Unbalance in monocyte subpopulations expressing CD11c and CD206 hallmarks metabolic dysfunction but has not yet been studied in response to sucralose. Our goal was to examine the effect of a single sucralose sip on serum insulin and blood glucose and the percentages of classical, intermediate, and nonclassical monocytes in healthy young adults subjected to an oral glucose tolerance test (OGTT). This study was a randomized, placebo-controlled clinical trial. Volunteers randomly received 60 mL water as placebo (*n* = 20) or 48 mg sucralose dissolved in 60 mL water (*n* = 25), fifteen minutes prior to an OGTT. Blood samples were individually drawn every 15 minutes for 180 minutes for quantifying glucose and insulin concentrations. Monocyte subsets expressing CD11c and CD206 were measured at -15 and 180 minutes by flow cytometry. As compared to controls, volunteers receiving sucralose exhibited significant increases in serum insulin at 30, 45, and 180 minutes, whereas blood glucose values showed no significant differences. Sucralose consumption caused a significant 7% increase in classical monocytes and 63% decrease in nonclassical monocytes with respect to placebo controls. Pearson's correlation models revealed a strong association of insulin with sucralose-induced monocyte subpopulation unbalance whereas glucose values did not show significant correlations. Sucralose ingestion decreased CD11c expression in all monocyte subsets and reduced CD206 expression in nonclassical monocytes suggesting that sucralose does not only unbalance monocyte subpopulations but also alter their expression pattern of cell surface molecules. This work demonstrates for the first time that a 48 mg sucralose sip increases serum insulin and unbalances monocyte subpopulations expressing CD11c and CD206 in noninsulin-resistant healthy young adults subjected to an OGTT. The apparently innocuous consumption of sucralose should be reexamined in light of these results.

## 1. Introduction

Noncaloric artificial sweeteners, including aspartame, acesulfame k, and sucralose, are food additives that preserve the taste of sweetness without increasing the calories of food and beverages [1]. For this reason, consumption of noncaloric artificial sweeteners is now widely spread among people from all ages and socioeconomic status worldwide [2, 3]. Nevertheless, a growing number of clinical and experimental studies have now suggested that noncaloric artificial sweeteners are linked to the development of metabolic abnormalities including insulin resistance and glucose intolerance, especially sucralose [4–8]. A seminal study demonstrated that ingestion of 48 mg sucralose significantly increases the serum values of glucose and insulin in morbidly obese subjects of both sexes subjected to a 75 g oral glucose tolerance test (OGTT) [9]. Similarly, overweight subjects that consumed sucralose prior to an OGTT exhibited a 1.2-fold elevation in the insulin peak as compared to placebo controls [10]. Therefore, the apparently innocuous effect of sucralose and others noncaloric artificial sweeteners on glucose and insulin homeostasis should be reexamined in light of this evidence.

It is now well accepted that low-grade systemic inflammation is a central player in obesity and contributes to the pathogenesis of metabolic disease, especially alteration of glucose and insulin homeostasis [11–13]. Besides being characterized by abnormally high levels of cytokines such as tumor necrosis factor alpha (TNF-alpha) and interleukin-1 beta (IL-1beta), low-grade systemic inflammation is accompanied by alteration in monocyte subpopulations [14]. In humans, circulating monocytes are sorted in three different subpopulations according to the cell surface expression of CD14 and CD16 [15]. Classical monocytes exhibit high CD14 levels and show no expression of CD16 (CD14^++^CD16^−^). The intermediate monocyte subpopulation shows high CD14 expression and also exhibit CD16 expression (CD14^++^CD16^+^) whereas nonclassical monocytes produce low CD14 levels and show expression of CD16 (CD14^+^CD16^+^) [15, 16]. Interestingly, the percentage of nonclassical monocytes has been shown to elevate in obese subjects that exhibit increased insulin resistance and metabolic syndrome [17, 18]. In the same sense, the classical monocyte subpopulation has been shown to increase in obese individuals and correlates with higher proportion of CD11c^+^ macrophages in the visceral adipose tissue (VAT) [19]. CD11c is a beta-2 integrin with prominent functions in cell adherence of monocytes and macrophages to vascular endothelial tissue and VAT [19, 20]. Furthermore, monocytes have been shown to express CD206 [21], a cell surface marker highly expressed in anti-inflammatory macrophages that is also associated with improved insulin sensitivity in both humans and mice [22, 23]. In this way, modulation of CD11c and CD206 expression in monocyte-macrophage lineage cells is crucial not only in typical immune functions such as cell migration and inflammation but also in obesity and insulin resistance [24]. Thus, imbalance in monocyte subpopulations expressing CD11c and CD206 hallmarks metabolic dysfunction but has not yet been studied in response to noncaloric artificial sweetener consumption such as sucralose.

The main goal of this study was to examine the effect of a single sucralose sip on the percentages of classical, intermediate, and nonclassical monocytes expressing CD11c and CD206 in healthy young adults subjected to an OGTT, while also exploring the possible relationship of monocyte subpopulations with changes in glucose and insulin homeostasis.

## 2. Materials and Methods

### 2.1. Subjects and Study Design

Forty-five healthy adult volunteers of both sexes with homeostasis model assessment (HOMA) values ≤3.8, aging between 18 and 35 years, who attended to the Department of Internal Medicine and the Laboratory for Proteomics and Metabolomics of the General Hospital of Mexico from September 2016 to April 2018 were included in the randomized, parallel-group, placebo-controlled clinical trial. All of the study participants provided written informed consent, previously approved by the Institutional Ethical Committee of the General Hospital of Mexico, which guaranteed that the study was conducted in rigorous adherence to the principles described in the 1964 Declaration of Helsinki and its posterior amendment in 2013. Subjects were excluded of the study if they had previous diagnosis of type 1 diabetes mellitus (T1D), type 2 diabetes mellitus (T2D), cardiovascular disease, acute or chronic liver disease, acute or chronic renal disease, cancer, endocrine disorders, infectious diseases, and inflammatory or autoimmune disease. We also excluded of the study to HIV-, HCV-, and HBV-seropositive patients, pregnant or lactating women, and individuals with anti-inflammatory, antiaggregant, antihypertensive, and immunomodulatory medication including nonsteroidal anti-inflammatory drugs. All participants included in the study had 8-10 hr overnight fasting before being subjected to the OGTT.

### 2.2. Oral Glucose Tolerance Test

The present study was a randomized, parallel-group, placebo-controlled clinical trial, where volunteers randomly drank 60 mL water as placebo (*n* = 20) or 48 mg sucralose dissolved in 60 mL water (*n* = 25), fifteen minutes prior to an OGTT. A regular “light” beverage available in the market approximately contains 48 mg sucralose. For this reason, we decided to use 48 mg sucralose dissolved in 60 mL water, as previously reported [9]. Each participant had up to three minutes to finish the sip of water or sucralose. Starting with oral glucose load at min zero, venous blood samples were drawn from all study subjects every 15 min for 180 min for quantifying the blood levels of glucose and insulin. Additional blood samples were also collected at -15 and 180 min for white blood cell isolation and characterization of monocyte subpopulations by flow cytometry.

### 2.3. Anthropometric and Biochemical Measurements

Body mass index (BMI), waist circumference, and blood pressure were measured in all study volunteers. Serum levels of insulin were measured in triplicate by the enzyme-linked immunosorbent assay (ELISA) following the manufacturer's instructions (Abnova Corporation, Taiwan). Serum levels of glucose were measured in triplicate by the glucose oxidase assay, following the manufacturer's instructions (Megazyme International, Ireland). The HOMA index was individually calculated by multiplying glucose concentration (mmol/L) by insulin concentration (mU/L) and then divided by 22.5. The cut-off point for HOMA index was established according to studies previously validated in a Mexican population [25]. Total cholesterol, low-density lipoproteins (LDL), high-density lipoproteins (HDL), and triglyceride levels were measured in triplicate by enzymatic assays following the manufacturer's instructions (Roche Diagnostics, Mannheim, Germany). Glycated hemoglobin (HbA1c), blood urea nitrogen (BUN), serum creatinine, and hematic biometry were determined by standard laboratory assays.

### 2.4. Flow Cytometry

Isolation of white blood cells was performed by centrifuging blood samples previously collected in tubes containing EDTA (Vacutainer™, BD Diagnostics, NJ, USA) at 1800 g for 10 min. Then, white blood cells were placed in 1.6 mL pyrogen-free Eppendorf tubes containing 1 mL ACK Lysing Buffer (Life Technologies, USA) and incubated at 4°C for 5 min. Afterward, cell suspension was centrifuged at 1800 g/4°C for 10 min and cell pellets washed twice with PBS 1x (Sigma-Aldrich, Mexico). After an additional centrifugation step and removal of the supernatant, cell pellets were resuspended in 50 *μ*L PBS 1x (Sigma-Aldrich, Mexico). Immediately after, 3 *μ*L Human TruStrain Reagent (BioLegend Inc., USA) was added to 2 × 10^5^ white blood cells and then incubated for 10 min at 4°C. Then, each cell suspension was incubated with anti-CD14 PE/Cy7, anti-CD16 FITC, anti-CD11c APC, and anti-CD206 PE (BioLegend Inc., USA) for 30 min at 4°C. Flow cytometry analysis was performed on a FACSCanto II flow cytometer by using the BD FACSDiva™ software 6.0 (BD Biosciences, Mexico), acquiring 1 × 10^5^ monocyte events per test in duplicate.

### 2.5. Gating Strategy for Flow Cytometry

White blood cells were gated for singlets on a forward scatter height/forward scatter area density plot. Afterward, areas corresponding to lymphocyte, polymorphonuclear leukocyte, and monocyte cell populations were clearly revealed and gated on a FSC-A/side scatter area plot. The monocyte gate was then selected for detection of living monocytes by using the Live/Dead Aqua Stain (Thermo Fisher Scientific Inc., USA) and posterior measurement of CD14, CD16, CD11c, and CD206 exclusively on this immune cell population. In this way, we deliberately excluded any other CD14-CD16-CD11c-CD206 signal coming from a different cellular source than monocytes. Monocyte subpopulations were then characterized according to the cell surface expression of CD14 and CD16 as follows: CD14^++^CD16^−^, classical monocytes; CD14^++^CD16^+^, intermediate monocytes; and CD14^+^CD16^+^, nonclassical monocytes.

### 2.6. Statistics

Normality of data distribution was estimated by the Shapiro-Wilk test. The Student *T*-test was used to compare the placebo and sucralose groups regarding age, BMI, waist circumference, systolic and diastolic blood pressure, blood glucose, glycated hemoglobin percentage, serum insulin, HOMA-IR, total cholesterol, LDL, HDL, triglycerides, blood urea nitrogen, serum creatinine, and hematic biometry, and data were expressed as mean ± standard deviation. The mean fluorescence intensity (MFI) of CD11c and CD206 as well as the percentages of classical, intermediate, and nonclassical monocytes at -15 and 180 min of the OGTT were analyzed using two-tailed 2-way ANOVA with correction for multiple comparisons by means of the Bonferroni multiple comparison test, and data were expressed as media ± standard deviation. Differences in the mean values of glucose and insulin at -15, 0, 15, 30, 45, 60, 75, 90, 105, 120, and 180 min of the OGTT, between volunteers enrolled in placebo or sucralose groups, were estimated by means of two-tailed 2-way ANOVA with correction for multiple comparisons using the Bonferroni multiple comparison test. Furthermore, the women/men proportion in placebo and sucralose groups was analyzed by means of the chi-squared test and data expressed as absolute values. Pearson's correlation coefficients were estimated for examining the statistical correlation of classical, intermediate, and nonclassical monocytes with glucose and insulin and expressed as coefficients (*r*) and *P* values. Differences were considered significant when *P* < 0.05. Statistical analyses were performed by means of the GraphPad Prism 6.01 software (GraphPad Software, La Jolla, CA 92037 USA).

## 3. Results

There were no differences between subjects receiving placebo (*n* = 20) or sucralose (*n* = 25) in terms of demographic, metabolic, and hematic characteristics, including gender, age, BMI, HOMA-IR, lipid profile, renal function, and monocytes per microliter of blood (Table 1).

**Figure 1:**
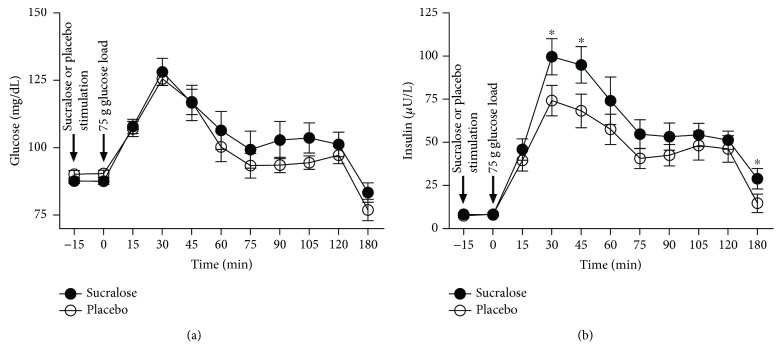
Blood levels of glucose and insulin in healthy young adults that received sucralose or placebo during an oral glucose tolerance test. Volunteers randomly received 60 mL water as placebo (*n* = 20) or 48 mg sucralose dissolved in 60 mL water (*n* = 25) 15 min prior to a 75 g oral glucose tolerance test (OGTT). Starting with glucose load at minute zero, venous blood samples were drawn from all study subjects every 15 min for 180 min for quantifying the blood levels of glucose and insulin. (a) Blood glucose did not show significant changes in subjects receiving placebo or sucralose all along the OGTT. (b) Serum insulin significantly increased at 30, 45, and 180 min in volunteers that received sucralose as compared to placebo controls. Timing of stimulation with sucralose, placebo, or glucose is shown on the graphic by black arrows. The placebo group is shown in open circles, whereas the sucralose group can be seen in closed circles. Data are expressed as media ± standard error. Significant differences between subjects receiving placebo or sucralose were estimated on each point of the OGTT by performing two-tailed 2-way ANOVA with correction for multiple comparisons by means of the Bonferroni multiple comparisons test. Significant differences are indicated by asterisks. Differences were considered significant when *P* < 0.05.

Blood glucose values showed no significant differences in subjects receiving placebo or sucralose all along the oral glucose tolerance test (Figure 1(a)). On the contrary, volunteers that received 48 mg sucralose prior glucose load showed a significant 1.3-fold increase in the serum levels of insulin at 30 min as compared to subjects drinking water as placebo (*P* = 0.041) (Figure 1(b)). At 45 min, subjects receiving sucralose exhibited a significant 1.4-fold elevation in serum insulin with respect to placebo controls (*P* = 0.046) (Figure 1(b)). At 180 min, subjects that received sucralose showed a significant 2-fold increase in the serum levels of insulin as compared to placebo controls (*P* = 0.048) (Figure 1(b)).

**Figure 2:**
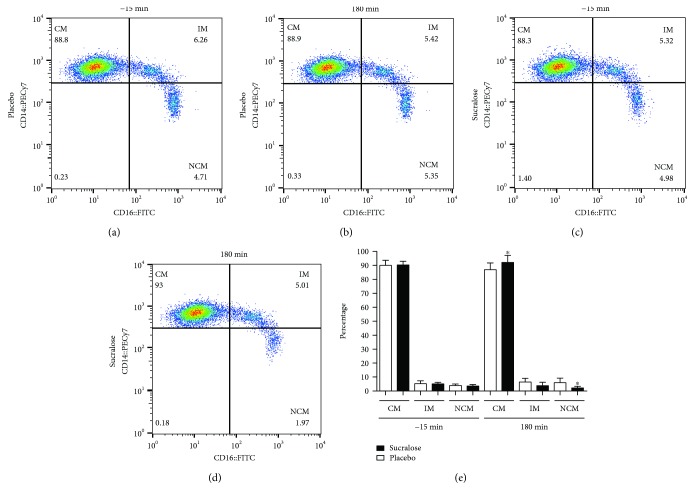
Percentages of classical, intermediate, and nonclassical monocytes in healthy young adults that received sucralose or placebo at the beginning and at the end of an oral glucose tolerance test. Representative flow cytometry dot plots showing the percentages of classical (CM), intermediate (IM), and nonclassical monocytes (NCM) in the placebo group at the beginning (a) and at the end (b) of the oral glucose tolerance test (OGTT). Representative dot plots showing the percentages of CM, IM, and NCM in the sucralose group at the beginning and at the end of the OGTT can be seen in (c) and (d), respectively. (e) As expected, quantification of monocyte subpopulation percentages showed no differences between placebo and sucralose groups at the beginning of the OGTT (-15 min). At 180 min, the CM percentage significantly increased whereas the NCM percentage decreased in volunteers that received 48 mg sucralose as compared to subjects that received water as placebo. No significant differences were seen in the IM percentage. The placebo group is shown in open bars, whereas the sucralose group can be seen in closed bars. Monocytes were gated on a CD14^+^CD16^+^ dot plot to identify monocyte subpopulations as follows: CD14^++^CD16^−^, classical monocytes; CD14^++^CD16^+^, intermediate monocytes; and CD14^+^CD16^+^, nonclassical monocytes. Data are expressed as media ± standard deviation. Significant differences between placebo and sucralose groups were estimated by performing two-tailed, 2-way ANOVA followed by the Bonferroni multiple comparisons test. Significant differences are indicated by asterisks. Differences were considered significant when *P* < 0.05.

Representative dot plots illustrating monocyte subpopulations in subjects that received placebo or sucralose are shown in Figure 2. At the beginning of the oral glucose tolerance test (-15 min), no differences were seen in the percentages of classical, intermediate, and nonclassical monocytes in subjects receiving placebo or sucralose (Figure 2(a) versus 2c, respectively). At the end of the experiment (180 min), the percentage of classical monocytes increased in subjects that received 48 mg sucralose as compared to placebo controls (Figure 2(d) versus 2b, respectively). In contrast, the nonclassical monocyte percentage was reduced in subjects exposed to sucralose with respect to placebo controls (Figure 2(d) versus 2b, respectively). Quantification of monocyte subpopulation percentages confirmed a significant 7% increase in the number of classical monocytes from subjects that received sucralose in comparison to controls receiving placebo (*P* = 0.0028) (Figure 2(e)). On the other side, the nonclassical monocyte percentage exhibited a significant 63% reduction in subjects exposed to sucralose with respect to placebo controls (*P* < 0.0001) (Figure 2(e)). No significant differences were found in the percentage of intermediate monocytes in subjects that received placebo or sucralose.

Analysis of Pearson's correlation coefficients showed no significant associations of monocyte subpopulations with blood glucose (Table 2). On the contrary, serum insulin showed a positive relationship with classical monocytes (*r* = 0.41, *P* = 0.02), whereas it also exhibited a strong inverse association with nonclassical monocytes (*r* = −0.42, *P* = 0.01) (Table 2).

**Figure 3:**
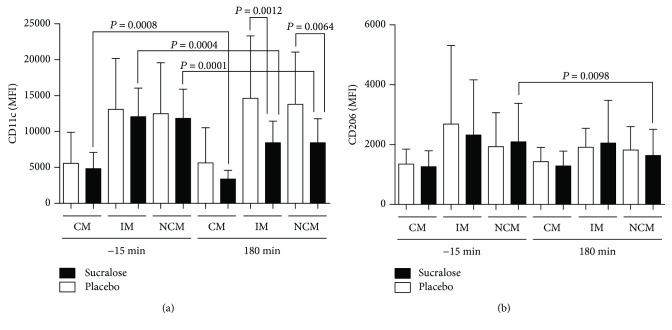
Cell surface expression of CD11c and CD206 in classical, intermediate, and nonclassical monocytes of healthy young adults that received sucralose or placebo at the beginning and at the end of an oral glucose tolerance test. (a) As expected, CD11c expression showed no differences between placebo and sucralose groups at the beginning (-15 min) of the oral glucose tolerance test (OGTT). At 180 min, CD11c expression significantly decreased in intermediate monocytes (IM) and nonclassical monocytes (NCM) of subjects that received 48 mg sucralose as compared to placebo controls. When comparing -15 and 180 min, classical monocytes (CM), IM, and NCM showed decreased CD11c expression in volunteers receiving sucralose. (b) CD206 expression showed no differences in subjects receiving placebo or sucralose at the beginning of the OGTT (-15 min). At 180 min, CD206 expression significantly decreased in the NCM subpopulation of subjects that received sucralose as compared to placebo controls. The placebo group is shown in open bars, whereas the sucralose group can be seen in closed bars. Monocytes were gated on a CD14^+^CD16^+^ dot plot to identify CD14^++^CD16^−^ classical monocytes, CD14^++^CD16^+^ intermediate monocytes, and CD14^+^CD16^+^ nonclassical monocytes and then measure the mean fluorescence intensity (MFI) of CD11c and CD206 on each monocyte subset. Data are expressed as media ± standard deviation. Significant differences between subjects receiving placebo or sucralose were estimated by performing two-tailed, 2-way ANOVA followed by the Bonferroni multiple comparisons test. Significant differences are indicated by asterisks. Differences were considered significant when *P* < 0.05.

At the beginning of the OGTT, CD11c expression in classical, intermediate, and nonclassical monocytes showed no significant differences in subjects receiving placebo or sucralose (Figure 3(a)). At 180 min, CD11c expression was significantly reduced in intermediate and nonclassical monocytes of subjects receiving sucralose as compared to placebo controls (*P* = 0.0012 and *P* = 0.0064, respectively) (Figure 3(a)). CD11c expression also exhibited a significant reduction in intermediate and nonclassical monocytes of subjects exposed to sucralose at the beginning (-15 min) and at the end (180 min) of the OGTT (*P* = 0.0004 and *P* = 0.0001, respectively) (Figure 3(a)). In classical monocytes, CD11c expression significantly diminished in the sucralose group at the beginning (-15 min) and at the end (180 min) of the OGTT (*P* = 0.0008) (Figure 3(a)).

At the beginning of the OGTT, CD206 expression in classical, intermediate, and nonclassical monocytes showed no significant differences in subjects that received placebo or sucralose (Figure 3(b)). At 180 min, CD206 expression was significantly reduced in nonclassical monocytes of volunteers that received sucralose as compared to placebo controls (*P* = 0.0098) (Figure 3(b)).

## 4. Discussion

Noncaloric artificial sweeteners are now consumed by millions of people from all ages, gender, and socioeconomic status around the globe [26]. However, sucralose and other noncaloric artificial sweeteners have been now linked to disturbances in glucose and insulin homeostasis in both animal models and humans [5–9]. For this reason, it is still of great relevance to keep characterizing the possible deleterious effects of sucralose on human metabolism in randomized, parallel-group, placebo-controlled clinical trials.

In this study, we found that one single 48 mg sip of sucralose, a sucralose amount that is contained in numerous “light” beverages available in the market, increases serum insulin but not glucose in age- and sex-matched healthy young adults subjected to an OGTT. These data appear contrary to previous information showing that a similar amount of sucralose is able to elevate both insulin and glucose in morbidly obese individuals receiving a glucose load [9]. Nevertheless, this apparently contradictory evidence should be examined in light of previous information showing that healthy young adults with no insulin resistance have the ability to increase insulin secretion and thus effectively decrease the excess of blood glucose [27, 28]. In contrast, numerous studies have consistently shown that nondiabetic morbidly obese subjects exposed to a glucose load can clearly increase insulin secretion without achieving blood glucose clearance due to a marked insulin resistance [29–32]. In this scenario, it is feasible to suppose that sucralose consumption may have differential effects on healthy young adults and morbidly obese subjects due to the presence of insulin resistance. In other words, sucralose ingestion may stimulate insulin secretion and, in this way, reduce glucose levels in healthy young adults but not morbidly obese subjects that show higher levels of insulin resistance and thus glucose intolerance. The fact that sucralose is able to stimulate directly insulin secretion has been previously reported in pancreatic beta cell lines and mouse islets [33], but remains elusive in humans. However, present results support the role of sucralose in promoting pancreatic insulin secretion in healthy young women and men that show normal insulin sensitivity, a probable phenomenon that needs to be confirmed in other human populations with different genetic background.

As mentioned before, low-grade activation of monocytes and macrophages has been shown to associate with the development of hyperinsulinemia, glucose intolerance, and insulin resistance [17–19, 34]. In this sense, it is well known that nutritive sweeteners such as sucrose or glucose exert the ability to increase TNF-alpha and IL-1beta expression and downregulate interleukin-10 (IL-10) production in human monocyte-derived macrophages *in vitro* [35, 36]. However, the effect of noncaloric artificial sweeteners on immune cells remains elusive. A previous study showed that exposure of human whole blood leukocytes to sucralose is able to suppress interleukin-6 (IL-6) and IL-10 secretion *in vitro*, even in the presence of phytohemagglutinin (PHA) or lipopolysaccharide (LPS) [37]. Likewise, the CD3^+^ T cell percentage has been shown to increase in Peyer's patches and lamina propria of mice receiving sucralose in drinking water [5]. Moreover, CD3^+^ T cells in Peyer's patches also showed elevation in TNF-alpha and interferon-gamma (IFN-gamma) production, accompanied by reduced expression of IL-10, which supports the role of sucralose in modulating immune cell activation [5]. Concurring with previous information, our findings show for the first time that a single sip of sucralose significantly increases the percentage of classical monocytes and reduces the nonclassical monocyte subpopulation in healthy young adults receiving a 75 g glucose load.

Another phenomenon captured in our study involves the possible mechanism by which sucralose exerts its effects on classical and nonclassical monocytes in healthy young adults. Sweet taste of sucralose and other caloric and noncaloric sweeteners is mediated by G protein-coupled receptors (GPCR) T1R1, T1R2, and T1R3 [38]. Sweet taste receptors were firstly described in the gut [39], enteroendocrine cells, and pancreas [40]. Nevertheless, T1R3 has been also indentified in mouse peritoneal macrophages [41]. Notably, *in vitro* exposure of T1R3 to trehalose (a disaccharide consisting of two molecules of glucose) has been shown to associate with suppression of TNF-alpha and IL-1beta expression in murine macrophages [41]. It is then feasible to speculate that sucralose may alter CD14 and CD16 expression via T1R3, which would lead to unbalance in the percentages of classical and nonclassical monocytes. However, we still need to measure sweet taste receptor expression on human monocyte subpopulations to draw major conclusions regarding this point.

Another possible mechanism by which sucralose may orchestrate dynamic changes in classical and nonclassical monocyte subsets involves the probable role of serum insulin. In our study, increase in serum insulin was importantly correlated with elevation of classical monocytes and reduction of nonclassical monocytes only in volunteers that drank sucralose but not placebo. Devevre and coworkers previously demonstrated that serum insulin is significantly associated with classical, intermediate, and nonclassical monocyte subpopulations of morbidly obese patients with insulin resistance [18]. Moreover, another study showed that insulin is able to induce protein kinase B (AKT) phosphorylation in both human classical and nonclassical monocyte subsets in a dose-dependent fashion *in vitro* [42]. Furthermore, Bunn and coworkers also reported that insulin boosts the palmitate-induced TNF-alpha and IL-6 expression in human monocytes *in vitro* [43]. Taking into account that (1) sucralose can directly stimulate pancreatic insulin secretion via T1R2 and T1R3 [33] and (2) insulin is able to regulate human monocyte activity [42, 43], it is then reasonable to suppose that serum insulin may directly modify the percentages of classical and nonclassical monocytes in subjects receiving sucralose but not placebo. Although plausible, it is a speculative scenario and further experimental studies should be performed with the aim of determining the exact mechanism by which sucralose affects human monocyte subpopulations *in vivo*.

In humans, monocyte subpopulations have been shown to exert different immune roles that are associated with expression patterns of cell surface molecules [16]. In this sense, classical and intermediate monocytes have been described to express CCR2 and CD11c and thus play pivotal roles in cell adhesion and migration [19, 44]. On the other hand, nonclassical monocytes have been shown to exert inflammatory actions within the circulation and progressive loss of CD206 expression on these immune cells is associated with enhanced proinflammatory capacity [21]. In this sense, our results show that sucralose consumption associates with low CD11c expression in all monocyte subsets and suggest alteration of the migratory capacity of these cells. However, such a hypothesis needs to be experimentally tested before drawing major conclusions regarding the effect of sucralose on the monocyte migratory capacity. To this respect, it has been previously reported that trehalose ingestion reduces CD11c expression in the colonic mucosa of mice treated with 2,4,6-trinitrobenzenesulfonic acid (TNBS), which in turn was associated with less infiltration of immune cells and improvement of intestinal inflammation [45]. However, to the best of our knowledge this is the first study reporting that sucralose consumption is able to decrease CD11c expression in human monocytes, a notion that may open new avenues to investigate the effect of sucralose on immune cells. In parallel, our findings also show that sucralose consumption associates with low expression levels of CD206 in nonclassical monocytes. Since CD206 is a cell surface marker related to the anti-inflammatory ability of mononuclear cells, it is thus feasible to speculate that sucralose ingestion may associate with increased proinflammatory capacity of human monocytes. As mentioned above, Rosales-Gomez and collaborators recently demonstrated that sucralose consumption exerts proinflammatory effects on mouse CD3^+^ T cells by rising TNF-alpha and IFN-gamma expression and reducing the expression of IL-10 [5]. Similarly, Bian and coworkers found increased expression of the proinflammatory markers TNF-alpha and nitric-oxide synthase 2 (NOS2) in the liver of mice receiving sucralose in the drinking water [46]. Present evidence concurs with our findings and supports the idea that sucralose may exert proinflammatory actions on nonclassical monocytes by decreasing CD206 expression. Nevertheless, we want to state that we still did not conduct any experimental *in vitro* study that firmly supports a direct role of sucralose on the anti-inflammatory activity of monocyte subsets and we are unable to draw solid conclusions regarding this topic at this time. Therefore, further *in vitro* studies are needed to characterize the possible effect of sucralose as a nonprototypic proinflammatory signal able to decrease CD206 expression in human monocytes.

It is worth mentioning that this study has some limitations including a possible participation of the sucralose sweet taste that may modify insulin secretion via the central nervous system as well the limited number of participants in each group. The amendment of these limitations (i.e., by using capsules containing sucralose) will bring more solid data to study the effect of sucralose on human metabolism and immunology.

## 5. Conclusions

This work demonstrates that a single sip of 48 mg sucralose increases the serum levels of insulin in age- and sex-matched non-insulin-resistant young adults subjected to an OGTT. Sucralose consumption was not only related to elevated levels of insulin but also increased proportion of classical monocytes and reduced percentage of nonclassical monocytes that in turn showed low expression levels of CD11c and CD206. Present results expand on the body of work that links sucralose consumption with unbalance of immune cell populations and alteration of insulin homeostasis. This work is relevant since the amount of sucralose studied here is contained in numerous “light” beverages available in the market and encourages further research focused on exploring the potential long-term impact of noncaloric artificial sweeteners on insulin metabolism and immune response in humans.

## Figures and Tables

**Table 1:** Demographic, metabolic, and hematic characteristics of the study population.

Parameters	Placebo	Sucralose	*P* value
Gender (W/M)	8/12	8/17	0.288
Age (years)	21.55 ± 2.18	22.36 ± 2.99	0.158
BMI (kg/m^2^)	24.58 ± 3.63	23.67 ± 2.88	0.177
Waist circumference (cm)	82.27 ± 8.44	78.57 ± 8.37	0.074
SBP (mmHg)	111.10 ± 8.16	113.70 ± 13.91	0.225
DBP (mmHg)	70.75 ± 5.77	71.16 ± 7.33	0.419
Blood glucose (mg/dL)	88.20 ± 6.65	89.96 ± 5.68	0.172
HbA1c (%)	5.26 ± 0.24	5.23 ± 0.19	0.497
Serum insulin (*μ*U/L)	7.94 ± 2.91	8.31 ± 2.82	0.335
HOMA-IR (a.u.)	1.75 ± 0.70	1.85 ± 0.65	0.298
Total cholesterol (mg/dL)	166.70 ± 31.21	168.60 ± 32.53	0.418
LDL (mg/dL)	102.10 ± 28.92	99.80 ± 26.60	0.393
HDL (mg/dL)	43.05 ± 10.60	44.40 ± 12.20	0.349
Triglycerides (mg/dL)	111.20 ± 58.10	118.20 ± 102.70	0.392
BUN (mg/dL)	22.40 ± 6.98	23.71 ± 5.95	0.249
Serum creatinine (mg/dL)	0.82 ± 0.13	0.78 ± 0.13	0.165
Hematocrit (%)	45.63 ± 3.53	44.04 ± 4.37	0.097
Total leukocytes (10^3^/*μ*L)	6.38 ± 1.49	6.05 ± 0.96	0.187
Monocytes (10^3^/*μ*L)	0.43 ± 0.11	0.39 ± 0.10	0.109
Monocytes (%)	6.94 ± 1.77	6.42 ± 1.33	0.134

Data are expressed as media ± standard deviation. The Shapiro-Wilk test was used to estimate normality in data distribution. Significant differences were estimated by means of performing the Student *T*-test with the exception of women/men proportion that was estimated by means of the chi-squared test. Differences were considered significant when *P* < 0.05. Abbreviations: W: women; M: men; BMI: body mass index; SBP: systolic blood pressure; DBP: diastolic blood pressure; HbA1c: glycated hemoglobin; HOMA-IR: homeostatic model assessment of insulin resistance; LDL: low-density lipoprotein; HDL: high-density lipoprotein; BUN: blood urea nitrogen; a.u.: arbitrary units.

**Table 2:** Statistical correlations of monocyte subpopulations with blood levels of glucose and insulin in placebo and sucralose groups.

	Placebo				Sucralose				
	-15		180		-15		180		
	*r*	*P*	*r*	*P*	*r*	*P*	*r*	*P*	
Classical monocyte (%)	0.24	0.12	0.35	0.06	0.19	0.18	0.14	0.25	Glucose
Intermediate monocyte (%)	-0.15	0.28	-0.25	0.13	-0.20	0.16	0.06	0.38	
Nonclassical monocyte (%)	-0.20	0.18	-0.24	0.14	-0.37	0.06	0.12	0.27	

Classical monocyte (%)	0.01	0.46	0.10	0.33	0.06	0.37	*0.41*	*0.02*	Insulin
Intermediate monocyte (%)	0.31	0.08	-0.21	0.17	0.24	0.12	-0.27	0.09	
Nonclassical monocyte (%)	-0.36	0.06	0.04	0.42	-0.29	0.07	*-0.42*	*0.01*	

Coefficients (*r*) and *P* values were calculated by the Pearson correlation model. The correlation level was considered significant when *P* < 0.05. Significant associations are marked in italic.

## Data Availability

The data used to support the findings of this study are available from the corresponding author upon request.
